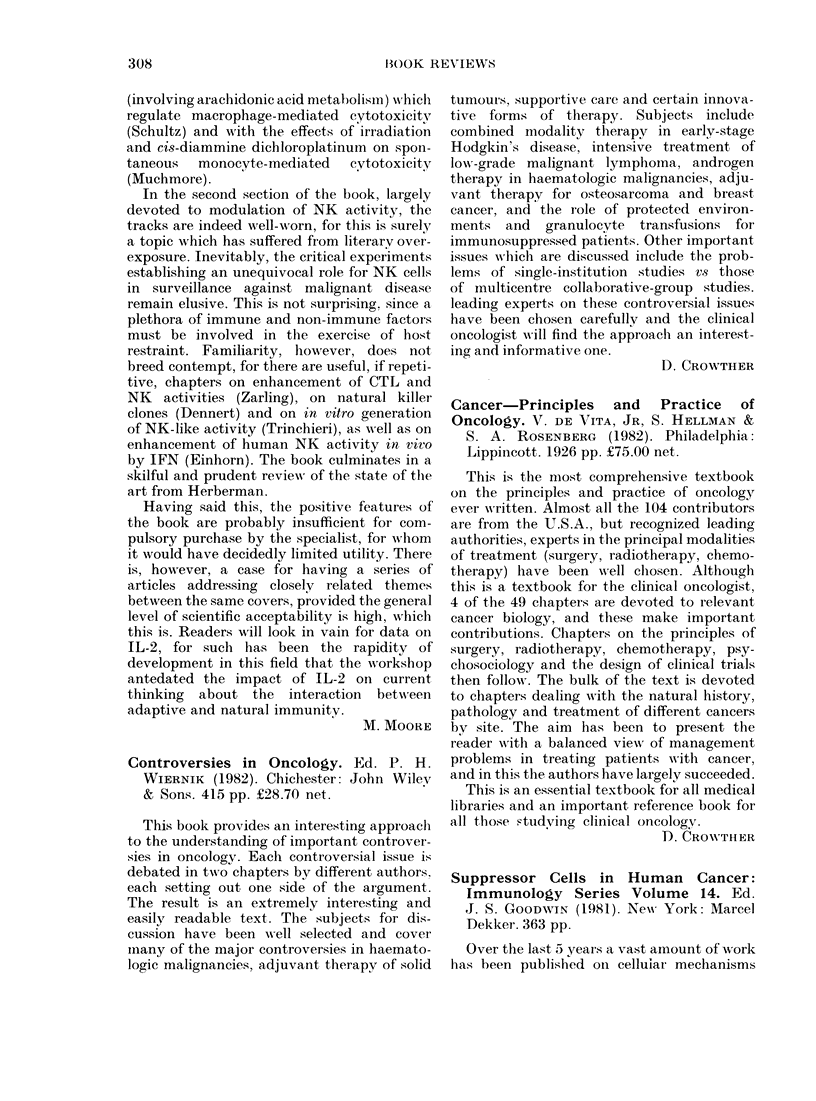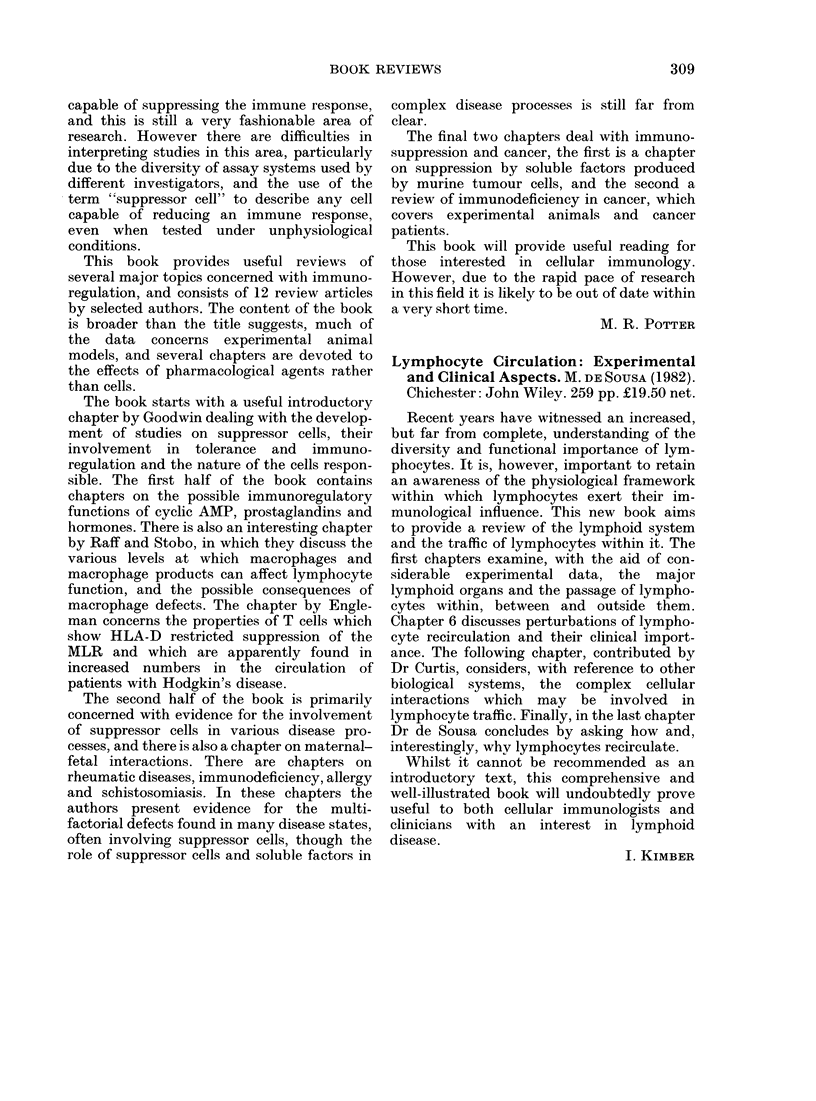# Suppressor Cells in Human Cancer: Immunology Series Volume 14

**Published:** 1982-08

**Authors:** M. R. Potter


**Suppressor Cells in Human Cancer: Immunology Series Volume 14.** Ed. J. S. GOODWIN (1981). New York: Marcel Dekker. 363 pp.

Over the last 5 years a vast amount of work has been published on celluiar mechanisms capable of suppressing the immune response, and this is still a very fashionable area of research. However there are difficulties in interpreting studies in this area, particularly due to the diversity of assay systems used by different investigators, and the use of the term "suppressor cell" to describe any cell capable of reducing an immune response, even when tested under unphysiological conditions.

This book provides useful reviews of several major topics concerned with immunoregulation, and consists of 12 review articles by selected authors. The content of the book is broader than the title suggests, much of the data concerns experimental animal models, and several chapters are devoted to the effects of pharmacological agents rather than cells.

The book starts with a useful introductory chapter by Goodwin dealing with the development of studies on suppressor cells, their involvement in tolerance and immunoregulation and the nature of the cells responsible. The first half of the book contains chapters on the possible immunoregulatory functions of cyclic AMP, prostaglandins and hormones. There is also an interesting chapter by Raff and Stobo, in which they discuss the various levels at which macrophages and macrophage products can affect lymphocyte function, and the possible consequences of macrophage defects. The chapter by Engleman concerns the properties of T cells which show HLA-D restricted suppression of the MLR and which are apparently found in increased numbers in the circulation of patients with Hodgkin's disease.

The second half of the book is primarily concerned with evidence for the involvement of suppressor cells in various disease processes, and there is also a chapter on maternal–fetal interactions. There are chapters on rheumatic diseases, immunodeficiency, allergy and schistosomiasis. In these chapters the authors present evidence for the multifactorial defects found in many disease states, often involving suppressor cells, though the role of suppressor cells and soluble factors in complex disease processes is still far from clear.

The final two chapters deal with immunosuppression and cancer, the first is a chapter on suppression by soluble factors produced by murine tumour cells, and the second a review of immunodeficiency in cancer, which covers experimental animals and cancer patients.

This book will provide useful reading for those interested in cellular immunology. However, due to the rapid pace of research in this field it is likely to be out of date within a very short time.

M. R. Potter